# Pauci-Immune Necrotizing and Crescentic Glomerulonephritis with Membranous Lupus Nephritis, Fifteen Years after Initial Diagnosis of Secondary Membranous Nephropathy

**DOI:** 10.1155/2015/120762

**Published:** 2015-10-08

**Authors:** Ryan Burkhart, Nina Shah, Michael Abel, James D. Oliver, Matthew Lewin

**Affiliations:** ^1^Department of Internal Medicine, William Beaumont Army Medical Center, 5005 N. Piedras Street, El Paso, TX 79920, USA; ^2^Department of Nephrology, William Beaumont Army Medical Center, 5005 N. Piedras Street, El Paso, TX 79920, USA; ^3^Department of Rheumatology, William Beaumont Army Medical Center, 5005 N. Piedras Street, El Paso, TX 79920, USA; ^4^Nephrology Service, Walter Reed National Military Medical Center, 8901 Rockville Pike, Bethesda, MD 20889, USA; ^5^ProPath Services, LLP, 1355 River Bend Drive, Dallas, TX 75247, USA

## Abstract

Renal involvement in systemic lupus erythematosus (SLE) is usually immune complex mediated and may have multiple different presentations. Pauci-immune necrotizing and crescentic glomerulonephritis (NCGN) refers to extensive glomerular inflammation with few or no immune deposits that may result in rapid decline in renal function. We report a case of a 79-year-old Hispanic male with a history of secondary membranous nephropathy (diagnosed by renal biopsy 15 years previously) who was admitted with acute kidney injury and active urinary sediment. P-ANCA titers and anti-myeloperoxidase antibodies were positive. The renal biopsy was diagnostic for NCGN superimposed on a secondary membranous nephropathy. A previous diagnosis of SLE based on American College of Rheumatology criteria was discovered via Veteran's Administration records review after the completion of treatment for pauci-immune NCGN. ANCAs are detected in 20–31% of patients with SLE. There may be an association between SLE and ANCA seropositivity. In patients with lupus nephritis and biopsy findings of necrotizing and crescentic glomerulonephritis, without significant immune complex deposition, ANCA testing should be performed. In patients with secondary membranous nephropathy SLE should be excluded.

## 1. Introduction

Pauci-immune necrotizing and crescentic glomerulonephritis (NCGN) refers to extensive glomerular inflammation with few or no immune deposits that may result in rapid decline in renal function if left untreated. Lupus nephritis (LN) can present with a NCGN. This often presents as a clinical syndrome of type 2 rapidly progressive glomerulonephritis (RPGN), pathologically consistent with class IV lupus nephritis and is immune complex mediated [1]. Often those patients have evidence of clinically or immunologically active lupus [2–5]. The first two cases of biopsy proven anti-neutrophil cytoplasmic antibody (ANCA) associated NCGN superimposed on a patient with class V LN were published in 1997 [5]. Since then this has remained a rare occurrence with three additional cases reported [3, 6, 7].

We describe a rare case of a patient with inactive SLE who presented with ANCA associated NCGN superimposed on class V LN fifteen years after his initial diagnosis of secondary membranous nephropathy.

## 2. Case Presentation

A 79-year-old Hispanic male presented to the emergency room with complaints of increased fatigue and decreased appetite. Fifteen years prior, he had presented with nephrotic range proteinuria (7.5 g/day on 24-hour collection) and underwent a renal biopsy showing secondary membranous glomerulopathy of unspecified etiology. Since the biopsy, his renal function was preserved and he was noted to have spontaneous remission of his proteinuria on prednisone without cytotoxic therapy. His other past medical history included mild dementia, hypertension, hypothyroidism, hyperlipidemia, gout, cerebral vascular disease, fatty liver, and alcohol abuse. Twelve years prior to his current presentation, his ANCA antibodies were negative. Six months prior, his serum creatinine was 114.92 *μ*mol/L (1.3 mg/dL). His medications were levothyroxine, allopurinol, sertraline, metoprolol tartrate, aspirin, galantamine, calcium/vitamin D, loratadine, vitamin E, and multivitamin. On presentation the blood pressure was 225/90 mmHg. The exam was significant for bilateral crackles on pulmonary exam and absence of lower extremity edema. Labs were significant for BUN of 32.84 mmol/L (92 mg/dL) and serum creatinine was 813.28 *μ*mol/L (9.2 mg/dL). Urinalysis was notable for 3+ proteinuria, 3+ blood, and specific gravity of 1.009. Urine sediment demonstrated 0–2 granular casts/hpf, 0-1 broad granular cast/lpf, and sheets of RBCs with >30% dysmorphic RBCs/hpf. Proteinuria was noted to be 3 g/day on a 24-hour collection. Serologies for HIV, hepatitis B, hepatitis C, and RPR were negative. Complement levels were normal. CRP was 2120.99 nmol/L (22.27 mg/dL), and ESR was 96 mm/hr. ANA was equivocal and anti-dsDNA antibodies were negative. Anti-Smith antibodies were negative. C-ANCA and anti-proteinase 3 antibodies were negative, as were anti-glomerular basement membrane (anti-GBM) antibodies. P-ANCA antibodies were positive with a 1 : 640 titer and anti-MPO antibodies were positive at 657 AU/mL (positive, >120 AU/mL). Chest X-ray showed small pleural effusions and patchy opacities bilaterally. Renal ultrasound noted normal parenchyma and no evidence of hydronephrosis or renal vein thrombosis.

Echocardiogram noted a preserved ejection fraction, moderate mitral stenosis, and elevated pulmonary artery pressures in the setting of a low normal central venous pressure. CT chest was consistent with chronic interstitial lung disease. Interstitial lung disease in combination with his mitral stenosis was likely contributing to his elevated pulmonary arterial pressures and pulmonary crackles on physical exam findings.

**Figure 1:**
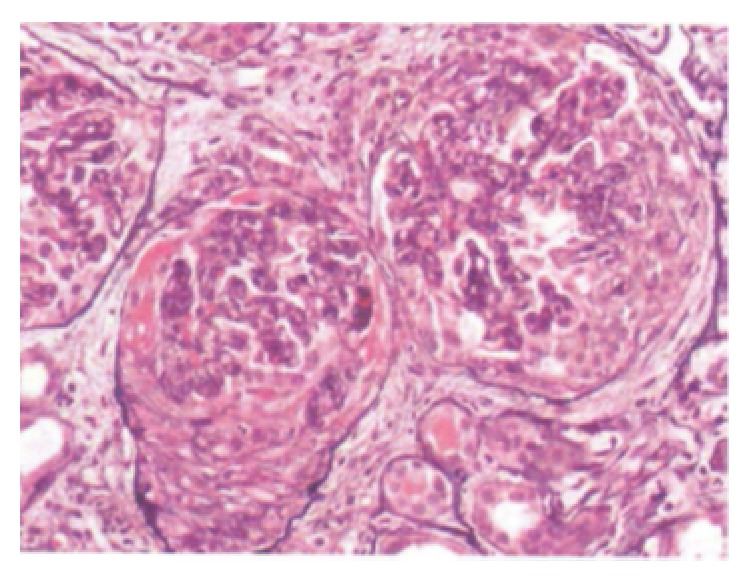
Light micrograph of crescentic glomerulonephritis with fibrinoid necrosis, showing mild mesangial expansion and minimal increase in cellularity without endocapillary proliferation. Subepithelial spikes were noted on the silver stain.

His blood pressure was treated with hydralazine and labetalol, and dialysis was initiated. A renal biopsy was performed and 39 glomeruli were obtained. Twelve out of 39 glomeruli were obsolescent, and 15 had cellular or fibrocellular crescents (Figure 1). Fibrinoid necrosis was present. There was mild increase in mesangial matrix but minimal hypercellularity and no endocapillary proliferation. The capillary walls were thickened, deposits were visible on Masson trichrome stain, and spikes were seen on Jones silver stain, consistent with a membranous glomerulopathy. The tubulointerstitium had inflammation with occasional eosinophils and mild interstitial fibrosis and tubular atrophy. No vasculitis was present in the vessels.

**Figure 2:**
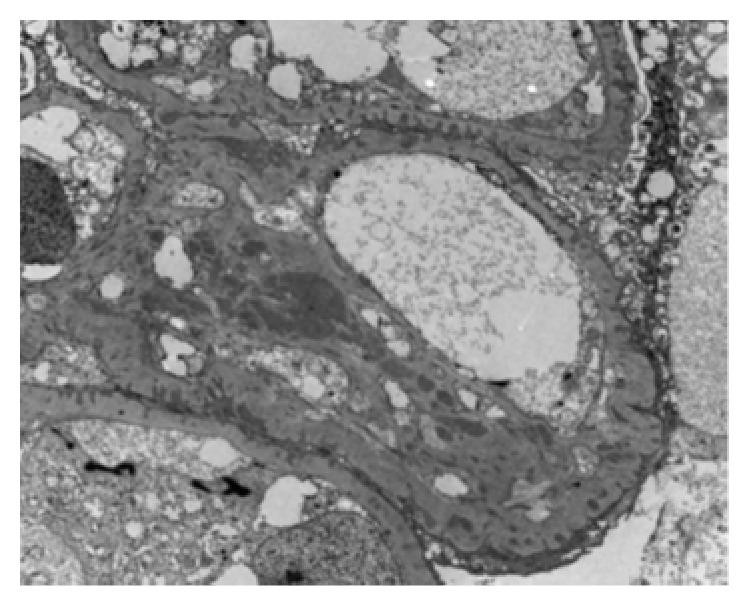
Electron microscopy showing extensive foot process effacement, numerous subepithelial deposits with GBM reaction-spike formation, and occasional enveloping of deposits. There are occasional mesangial deposits. Fibrin deposition was noted within Bowman's capsule.

Immunofluorescence was positive for IgG (3+), IgM (trace), C3 (3+), kappa (2+), and lambda (3+) in the mesangium and glomerular capillary wall. C1q was negative. There was segmental staining for fibrinogen (3+) in Bowman's capsule. Electron microscopy showed extensive foot process effacement and numerous subepithelial deposits with spike formation and occasional enveloping of deposits consistent with stage 3 membranous nephropathy (Figure 2). There were occasional mesangial deposits but no tubuloreticular inclusions or tubular basement membrane deposits.

Glomerular basement membrane immunofluorescent histology evaluation for IgG subclasses 1–4 was positive only for IgG2 which was strongly suggestive of secondary membranous nephropathy. Staining for anti-phospholipase A2 receptor (anti-PLA2R) antibodies was negative.

Treatment was initiated for renal-limited NCGN with corticosteroids (1 gram of methylprednisolone, 1 gm intravenously daily for three days, followed by prednisone 60 mg orally daily with gradual taper), plasmapheresis (seven treatments, one plasma volume every other day), and rituximab (375 mg/m^2^ once weekly for four doses). Despite therapy, renal recovery was insufficient to withdraw dialysis. On clinical followup at sixteen months the patient remained dialysis dependent but free of systemic manifestations of vasculitis.

A diagnosis of SLE from fifteen years ago based on American College of Rheumatology (ACR) and Systemic Lupus International Collaborating Clinics (SLICC) criteria was discovered via Veteran's Administration records review after the completion of treatment for pauci-immune NCGN.

## 3. Discussion

The patient had a history of secondary membranous nephropathy of unspecified etiology since 1998. Prior work-up at that time was negative for viral hepatitis and negative for RPR, with no known history of drug induced membranous nephropathy or malignancy. ANCA antibodies were negative three years after the initial renal biopsy but were not checked at the time of the initial diagnosis of secondary membranous nephropathy. In the fifteen years prior to his presentation, the proteinuria had resolved spontaneously without cytotoxic therapy.

A diagnosis of SLE was discovered via Veteran's Administration records review after the completion of treatment for pauci-immune NCGN. ANA titer with speckled pattern of 1 : 2560 was noted in 2001. There is prior dermatologist documentation of photosensitivity and skin biopsy report consistent with cutaneous lupus. He also had prior nephrotic range proteinuria documented with secondary membranous nephropathy of unspecified etiology. He met 4 of the 11 criteria diagnostic for systemic lupus erythematosus based on the prior ACR criteria. Subsequent discussion with the pathologist in conjunction with the above history confirmed that the patient's secondary membranous nephropathy would be consistent with International Society of Nephrology (ISN) Class V lupus nephritis. He would therefore also meet criteria for the diagnosis of SLE based on the Systemic Lupus International Collaborating Clinics (SLICC) criteria. We suspect that at some point in time he likely developed synovitis, probably from his underlying SLE, and given his positive low titer rheumatoid factor, was given the diagnosis of rheumatoid arthritis. His overall picture would fit better with SLE as well given his lack of erosive joint disease. It is well described that about a third of patients with SLE will have positive rheumatoid factor.

He was conjectured to have either renal limited vasculitis or early microscopic polyangiitis as he had no other systemic involvement. Given the severity of his disease, initial decision was to treat him with pulse methylprednisolone of 1 gram for three days followed by prednisone (1 mg/kg) with gradual taper and oral cyclophosphamide. Rituximab has shown evidence of noninferiority in randomized controlled trials [8, 9] and possibly reduces relapse among patient with renal vasculitis. KDOQI guidelines also note that rituximab may be used as an alternative [10]. Notably one critique for the use of rituximab is that it has not been well studied in patients with severely elevated creatinine (>354 *μ*mol/L [>4.0 mg/dL]) as they were excluded from the RAVE trial. Our patient presented with a serum creatinine of 813.28 *μ*mol/L (9.2 mg/dL); however, there was concern for the increased risk of adverse effects, particularly leukopenia and infection with cyclophosphamide, given the patient's age, baseline comorbidities, transition to oliguric state, and anemia. Rituximab was therefore used as the induction agent utilizing the RAVE trial protocol [9]. Prior data has suggested that the treatment of severe renal vasculitis with the use of plasma exchange [11, 12] may be of benefit to decrease the progression to ESRD. Plasma exchange was used as an adjunctive therapy prior to rituximab. Seven sessions of plasma exchange were performed every other day for the first two weeks as assigned in the MEPEX trial [12]. Given that the patient showed no evidence of renal recovery on followup nor manifestations of systemic vasculitis, his prednisone was tapered and no further immunologic agents were started.

The patient did not regain sufficient renal function to become dialysis independent. This was not all together unexpected given the patient's serum creatinine of 813.28 *μ*mol/L (9.2 mg/dL) on presentation, the mild interstitial fibrosis, and the fact that 12 of 39 glomeruli were obsolescent on renal biopsy. Serum creatinine at time of biopsy is an important predictor of progression to ESRD [13]. On clinical follow-up at sixteen months, the patient had not displayed evidence of systemic vasculitis despite remaining persistently anti-MPO antibody positive. Notably, since the patient's hospitalization, additional follow-up data has been published from the RAVE study at 18 months which noted sustained benefit from single course of induction therapy with rituximab [14], though our patient (who was anti-MPO antibody positive) may be at lower risk for relapse given that C-ANCA and anti-proteinase 3 antibody positivity is at higher risk for relapse [15].

When crescents and necrosis are noted superimposed on membranous nephropathy, the possibility of a mixed ISN/RPS class V LN with class III or IV LN may be considered. The renal biopsy noted minimal hypercellularity which would indicate that the necrotic lesions were “pauci-immune” in nature. In addition, the cellularity was limited to the mesangial areas without significant endocapillary proliferation and the degree of crescents and necrosis was out of proportion to the proliferative changes and the amount of immune complex deposits.

ANCA associated NCGN has been described to occur superimposed on primary [13, 16] and secondary membranous nephropathy [17] and has been reported with multiple classes of LN (at least classes II–V) [2–4] with different manifestations of systemic vasculitis. Since the first two cases of ANCA associated NCGN superimposed on class V LN in 1997 only three additional cases have been reported [3, 6, 7]. While there appears to be an association between SLE and ANCA positivity, it is unclear if there is a pathophysiologic mechanism for class V LN nephropathy resulting in NCGN. Our patient had documented negative ANCA antibodies twelve years prior and it is uncertain when the ANCA antibodies actually became positive, or if they were drug induced (i.e., allopurinol). Anti-MPO antibodies can be also be induced with hydralazine which was used for inpatient management of his hypertension. It would be unlikely to develop after two doses of hydralazine. Additionally he was in renal failure prior to the initial administration of the hydralazine.

Review of the literature has noted two case reports of NCGN that manifested or “transformed” years after a diagnosis of idiopathic membranous nephropathy. James et al. in 1995 [18] described a 67-year-old male with idiopathic membranous nephropathy diagnosed fifteen years prior to his presentation with anasarca and AKI in setting of recent CABG, TURP, and urinary tract infection. Repeat renal biopsy showed crescentic GN and allergic interstitial nephritis.

Kwan et al. in 1991 [19] described a 31-year-old male idiopathic membranous nephropathy with persistent nephrotic range proteinuria despite treatment with steroids. Seven years later he presented clinically with rapidly progressive glomerulonephritis with biopsy noting crescentic GN. Both of these patients were ANCA and anti-GBM negative. Our patient presented with P-ANCA associated NCGN superimposed on class V LN, fifteen years after the initial diagnosis of secondary membranous nephropathy, which to the best of our knowledge has not yet been described.

## 4. Conclusion

There appears to be an association between SLE and ANCA seropositivity [20, 21]; however, the number of cases reported with class V LN and ANCA associated GN is small. In patients with lupus nephritis and biopsy findings of necrotizing and crescentic glomerulonephritis, without significant subendothelial immune complex deposition, endocapillary proliferation, or immunologic evidence of active SLE, ANCA testing should be considered. In patients with membranous nephropathy and renal biopsy suggestive of a secondary process, work-up must include the exclusion of underlying SLE.
